# Investigation of the Possible Allostery of Koumine Extracted From *Gelsemium elegans* Benth. And Analgesic Mechanism Associated With Neurosteroids

**DOI:** 10.3389/fphar.2021.739618

**Published:** 2021-10-04

**Authors:** Bojun Xiong, Wenbing You, Yufei Luo, Guilin Jin, Minxia Wu, Ying Xu, Jian Yang, Huihui Huang, Changxi Yu

**Affiliations:** ^1^ Department of Pharmacology, School of Pharmacy, Fujian Medical University, Fuzhou, China; ^2^ Key Laboratory of Gastrointestinal Cancer (Fujian Medical University), Ministry of Education, School of Basic Medical Sciences, Fujian Medical University, Fuzhou, China; ^3^ Fujian Key Laboratory of Drug Target Discovery and Structural and Functional Research, School of Pharmacy, Fujian Medical University, Fuzhou, China; ^4^ Public Technology Service Center, Fujian Medical University, Fuzhou, China

**Keywords:** koumine, allostery, translocator protein 18 kDa, probe dependence, neurosteroid

## Abstract

Translocator protein 18 kDa (TSPO) is an evolutionarily conserved 5-transmembrane domain protein, and has been considered as an important therapeutic target for the treatment of pain. We have recently reported the *in vitro* and *in vivo* pharmacological characterization of koumine as a TSPO positive allosteric modulator (PAM), more precisely ago-PAM. However, the probe dependence in the allostery of koumine is an important question to resolve, and the possible analgesic mechanism of koumine remains to be clarified. Here, we report the *in vivo* evaluation of the allostery of koumine when orthosteric ligand PK11195 was used and preliminarily explore the possible analgesic mechanism of koumine associated with neurosteroids. We find that koumine is an ago-PAM of the PK11195-mediated analgesic effect at TSPO, and the analgesic mechanism of this TSPO ago-PAM may be associated with neurosteroids as the analgesic effects of koumine in the formalin-induced inflammatory pain model and chronic constriction injury-induced neuropathic pain model can be antagonized by neurosteroid synthesis inhibitor aminoglutethimide. Although our results cannot fully clarify the allosteric modulatory effect of koumine, it further prove the allostery in TSPO and provide a solid foundation for koumine to be used as a new clinical candidate drug to treat pain.

## Introduction

*Gelsemium elegans* Benth. is indigenous to Southeast Asia, particularly China (Jin et al., 2014), and was first recorded in the earliest extant pharmaceutical monograph Divine Farmer’s Classic of Materia Medica (Shen Nong Ben Cao Jing) in China. In view of its obvious analgesic effect, the roots and leaves of *G. elegans* Benth. have been utilized orally or in dressings by the Hani ethnicity in the Naban River Watershed National Nature Reserve, Yunnan, China, for the treatment of bone fracture, stomachache and kidney pain (Ghorbani et al., 2011). Alkaloids are the main active ingredients of *G. elegans* Benth. Early clinical studies showed that the parenteral solution of crude alkaloid extraction has a significant analgesic effects on cancerous pain and colic caused by visceral smooth muscle spasm, with the analgesic efficiency reaching 90% (Chen, 1984). However, its further clinical use was restricted by its narrow therapeutic index. Koumine is the most abundant alkaloid in *G. elegans* Benth. with low toxicity (Jin et al., 2014), our previous series of studies showed that koumine has potent analgesic effect and no morphine-like tolerance or dependence. Therefore, it is of great value to develop koumine as a new candidate drug for pain treatment (Xu et al., 2012; Ling et al., 2014; Qiu et al., 2015; Xiong et al., 2017; Jin et al., 2018a; Jin et al., 2018b; Jin et al., 2021).

Translocator protein 18 kDa (TSPO) is an evolutionarily conserved 5-transmembrane domain protein previously known as peripheral benzodiazepine receptor (Papadopoulos et al., 2006). Genetic knockout studies have shown that although TSPO is not critical for the maintenance of baseline adrenal and gonadal steroidogenesis (except under stress or in aging), it can impair the total steroidogenic output (Banati et al., 2014; Morohaku et al., 2014; Tu et al., 2014; Fan et al., 2015; Owen et al., 2017; Barron et al., 2018). Given that TSPO ligands have important therapeutic effects in inflammatory and neuropathic pain (DalBo et al., 2004; Wei et al., 2013; Liu et al., 2016), TSPO is considered an important therapeutic target for the treatment of pain. In recent years, several studies have implied that there may be allostery in TSPO (Narlawar et al., 2015; Jaipuria et al., 2017; Rojas et al., 2018), however, further investigations are still needed.

We have recently reported the *in vitro* and *in vivo* pharmacological characterization of koumine as a TSPO positive allosteric modulator (PAM), more precisely ago-PAM, and further demonstrated the allostery in TSPO. Although the allosteric mechanisms of koumine was still unknown, our findings indicated that TSPO PAM may be a novel avenue for the treatment of inflammatory and neuropathic pain (Xiong et al., 2021). Notably, the allosteric interaction between an allosteric ligand and a protein is dependent on the orthosteric ligand (probe) used, that is probe dependence (Wootten et al., 2013). Our previous work focused on Ro5-4864 (one of the classical TSPO orthosteric ligands), the magnitude of the allosteric effect between koumine and TSPO when other TSPO orthosteric ligands were used remains to be examined. In addition, the results of our previous *in vitro* experiments showed that koumine did not show any allosteric modulatory effect in the synthesis of neurosteroid (Xiong et al., 2021). However, considering that koumine is a high-affinity ligand of TSPO and that the analgesic effect of TSPO ligands such as Ro5-4864 can be antagonized by neurosteroid synthesis inhibitor *in vivo* (Wei et al., 2013), whether koumine exerts analgesic effects *in vivo* through neurosteroidogenesis is also worthy of further study.

Given these findings, the aim of the current study was to evaluate the allosteric modulatory effect of koumine when the orthosteric ligand PK11195 was used and preliminarily explore the possible analgesic mechanism of koumine associated with neurosteroids. First, the effect of koumine on the analgesic effect of PK11195 *in vivo* was explored. Then, the possible analgesic mechanism of koumine was explored in the presence of a neurosteroidogenesis inhibitor in two pain models: the formalin-induced inflammatory pain model and the chronic constriction injury (CCI) model of neuropathic pain.

## Materials and Methods

### Chemicals and Reagents

The dried rhizome of *G. elegans* Benth. was obtained from Bozhou Guotao Pharmaceutical Co., Ltd., Anhui (China). Koumine (purity >99%, UPLC) was isolated from *G. elegans* Benth. using pH-zone-refining countercurrent chromatography and the chemical identity of koumine was verified by MS and NMR, as described previously (Su et al., 2011). PK11195, Ro5-4864 and aminoglutethimide (AMG) were purchased from Sigma-Aldrich (United States).

### Animals

Male ICR mice and male Sprague-Dawley rats (20 ± 2 g and 140 ± 10 g, respectively) were provided by the Beijing Vital River Laboratory Animal Technology Co., Ltd., China. A 12 h light/dark cycle with controlled temperatures of 20–26°C and 40–70% humidity was maintained in the barrier housing facility. Food and water were available *ad libitum* throughout the study period. All experimental procedures were performed according to the guidelines of the National Institutes of Health Guide for the Care and Use of Laboratory Animals with approval from the Committee of Ethics of Fujian Medical University, China.

### Formalin Test

The formalin test was carried out according to Dubuisson and Dennis (1977), with minor modifications. In brief, adult male ICR mice were individually placed in a transparent plastic box for adaptation, at least 30 min prior to injection of formalin. Each animal received a s.c. injection of 10 μl of 5% formalin solution into the plantar surface of the right hindpaw. The total licking time of the right hindpaw at 0–5 min (phase I) and 11–60 min (phase II) was recorded. PK11195 and koumine were injected s.c. 30 min or 40 min prior to formalin administration, respectively. To assess the interaction between koumine and PK11195, separate groups of mice were administrated koumine or vehicle and 10 min later PK11195 were given. For the antagonist study, i.p. AMG was administered 40 min before koumine, PK11195 or Ro5-4864 injection, and 70 min prior to formalin injection. PK11195, Ro5-4864 and AMG were dissolved in DMSO and diluted in sterile physiological saline (0.9% NaCl) to the appropriate concentration, and the final concentration of DMSO injected into animal was 5%. Koumine was dissolved in sterile physiological saline (0.9% NaCl) directly. The mock vehicle group was injected with 5% DMSO in saline.

### Chronic Constriction Injury Model

The rat model of neuropathic pain by unilateral ligation of sciatic nerve was performed in rats as described earlier (Bennett and Xie, 1988). Briefly, SD rats were anesthetized by isoflurane delivered using an anesthetic machine. The right common sciatic nerve was dissected, exposed, and ligated at the level of the midthigh using 4 chromic gut (4-0) ties, separated by a 1 mm interval. After ligation, the wound was sutured and the rats were allowed to recover. With the sciatic nerve ligated rats, only those with marked unilateral allodynia to mechanical stimulation (hind limb withdrawal thresholds in the ipsilateral side/contralateral side <0.75) and with no major motor impairment were selected for further studies. The withdrawal thresholds of ipsilateral hind limbs were measured using the procedures described below. On the 9th day after surgery, i.p. AMG was administered 40 min before Ro5-4864 or koumine injection, and the mechanical withdrawal threshold (MWT) was determined 50 min after the last injection. Ro5-4864 and AMG were dissolved in DMSO and diluted in sterile physiological saline (0.9% NaCl), and the final concentration of DMSO was 5%. Koumine was dissolved in sterile physiological saline (0.9% NaCl) directly, and then diluted to the specified concentration. The mock vehicle group was injected with 5% DMSO in saline.

### Measurement of Mechanical Allodynia

To assess mechanical allodynia, a 2390 Model electronic von Frey apparatus (IITC Life Science Inc., Woodland Hills, CA, United States) was used to determine ipsilateral hind limb withdrawal threshold evoked by stimulation of the hind paw as described by Mitrirattanakul et al. (2006) with minor modifications. Briefly, rat stood on a metal grid, the center of the hind paw was stimulated using the von Frey filament applied up to a maximum strength of 55 g or until the point of paw withdrawal. The threshold at which withdrawal occurred was automatically registered. The triplicate measurements were made at an interval of approximately 5 min, and the mean of these threshold values for each hind paw was used.

### Data and Statistical Analysis

Data are presented as the mean ± SEM and were analyzed using SPSS software (version 19.0, SPSS Inc.). The comparison between the two groups was analyzed by independent samples *t*-test, and multiple comparisons were compared by one-way ANOVA followed by LSD post hoc test. Statistical significance for all of the analysis was determined at *p* < 0.05.

## Results

### Koumine Augments PK11195-Mediated Analgesic Effects in a Formalin-Induced Inflammatory Pain Model

**FIGURE 1:**
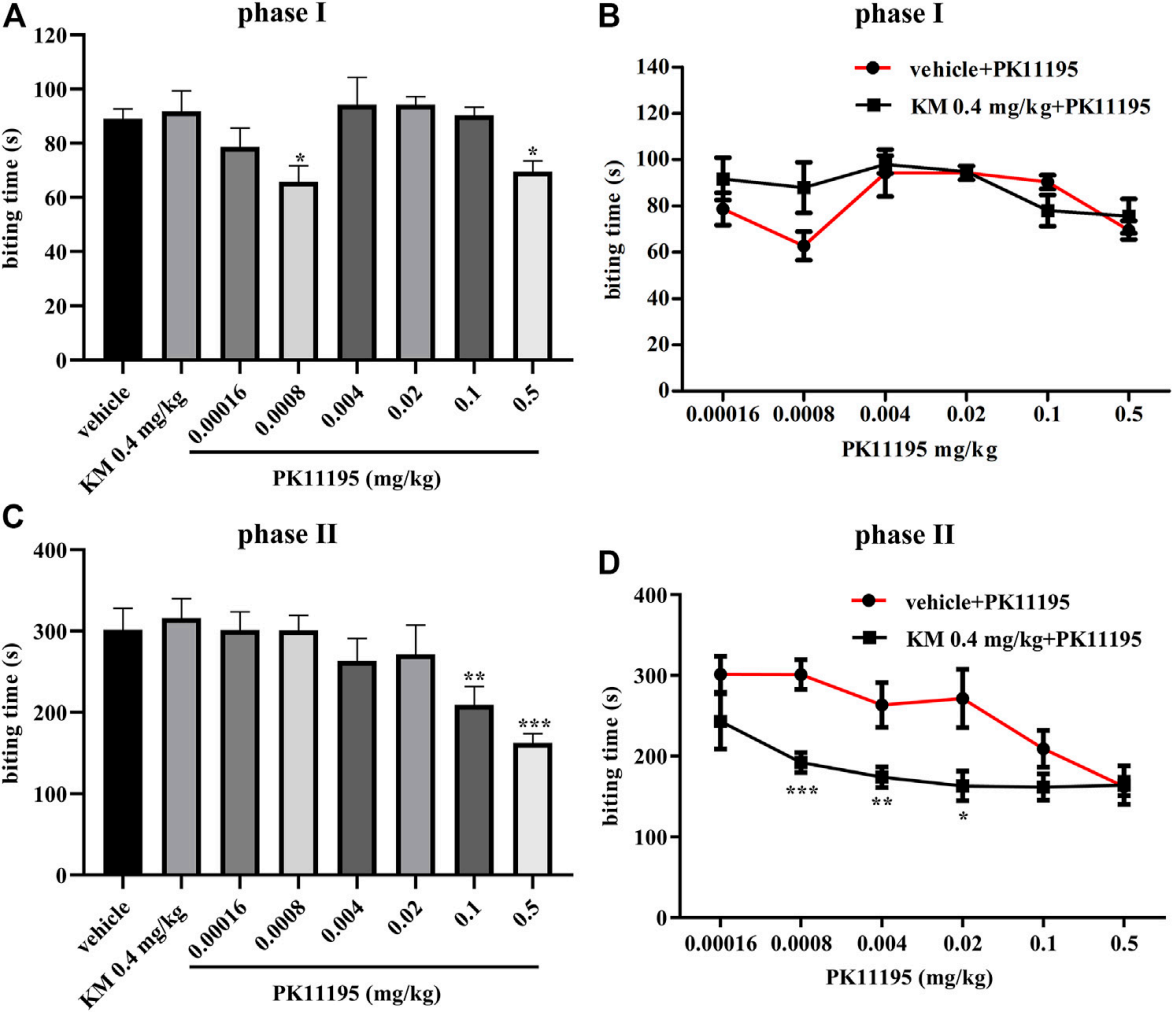
Koumine augments PK11195-mediated analgesic effects in the formalin test in mice. **(A, C)** Dose response effects of koumine and PK11195 on phase I **(A)** and phase II **(C)** in the formalin test. **(B, D)** Dose response curves for PK11195 in the absence or presence of koumine (0.4 mg/kg) on phase I **(B)** and phase II **(D)** in the formalin test. PK11195 and koumine were injected s.c. 30 min or 40 min prior to formalin administration, respectively. To assess the interaction between koumine and PK11195, separate groups of mice were administrated koumine or vehicle and 10 min later PK11195 were given. Abbreviations: KM, koumine. Data are represented as the mean ± SEM, *p < 0.05, **p < 0.01 and ***p < 0.001 vs. the corresponding vehicle group. The comparison between the two groups was analyzed by independent samples *t*-test, and multiple comparisons were compared by one-way ANOVA followed by LSD post hoc test. Each group consisted of 7–10 mice.

To evaluate the probe dependence of koumine, the formalin-induced inflammatory pain model was selected. According to the preliminary experimental results of our research group, koumine produced a dose-dependent antinociceptive effect in phase II, 0.4 mg/kg koumine is ineffective on its own but can significantly augment the Ro5-4864-mediated analgesic effect, indicating that koumine is a PAM of Ro5-4864-mediated analgesic effect at TSPO, more precisely ago-PAM, that is, allosteric modulators exhibit PAM effects at low concentrations, and can also exert their effects alone at high concentrations, showing allosteric agonist effects (Schwartz and Holst, 2007; Garai et al., 2018; Xiong et al., 2021). Therefore, we also choose 0.4 mg/kg koumine for the experiment in this manuscript. Figure 1 shows that there were significant among-group differences in the biting time in phase I and phase II (phase I: F_7, 66_ = 3.227, *p* = 0.005, Figure 1A; phase II: F_7, 67_ = 4.913, *p* < 0.001, Figure 1C). Post hoc LSD multiple comparison tests revealed that PK11195 produced a significant antinociceptive effect in phase I and phase II (phase I: *p* = 0.010 for 0.0008 mg/kg PK11195, *p* = 0.026 for 0.5 mg/kg PK11195, respectively; phase II: *p* = 0.008 for 0.1 mg/kg PK11195, *p* < 0.001 for 0.5 mg/kg PK11195, respectively), and the analgesic effect in phase I is not dose-dependent, which was consistent with the previous results of our and other research groups (DalBo et al., 2004; Xiong et al., 2021). Simultaneously, there was no analgesic effect of 0.4 mg/kg koumine (phase I: *p* = 0.763, phase II: *p* = 0.681, Figures 1A,C). When 0.4 mg/kg koumine was combined with PK11195, koumine prominently augmented the 0.0008–0.02 mg/kg PK11195-mediated analgesic effect in phase II but have no significant effect on phase I (phase II: *p* < 0.001 for 0.0008 mg/kg PK11195, *p* = 0.009 for 0.004 mg/kg PK11195 and *p* = 0.013 for 0.02 mg/kg PK11195, Figures 1B,D).

### Koumine-Mediated Analgesic Effects *In Vivo* can be Prevented by Aminoglutethimide

**FIGURE 2:**
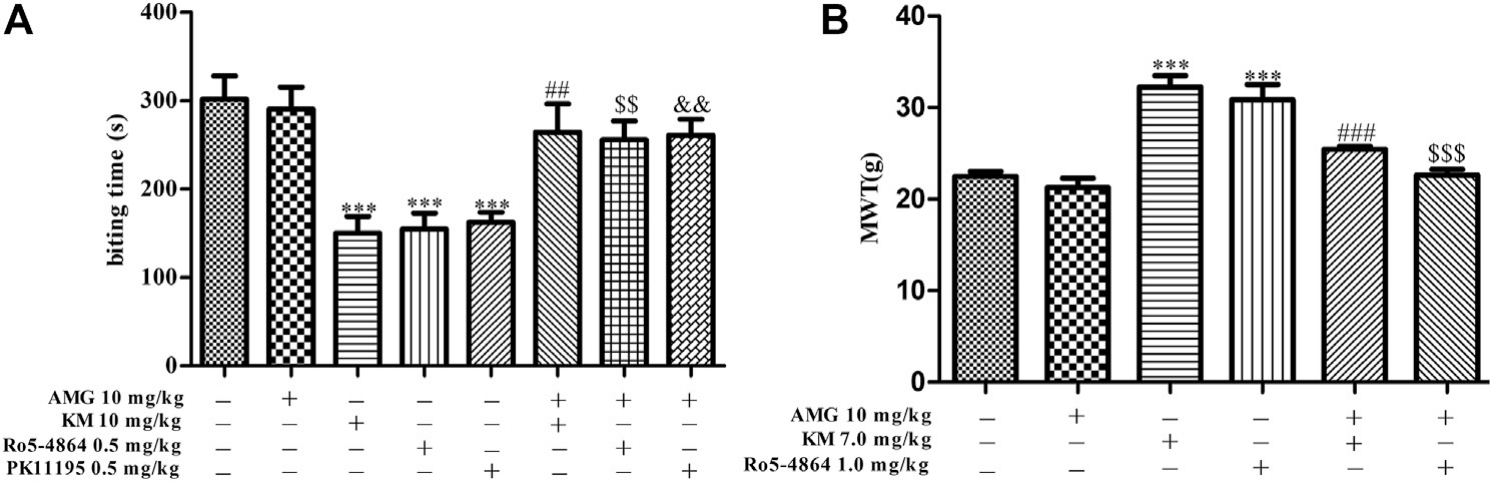
Koumine-mediated analgesic effects *in vivo* can be prevented by AMG. **(A)** Application of AMG (10 mg/kg, i.p.) 40 min before s.c. injection of the TSPO ligand abolished the anti-inflammatory pain effects in the formalin test in mice. **(B)** Application of AMG (10 mg/kg, i.p.) 40 min before the koumine (s.c.) or Ro5-4864 (i.p.) injection abolished the anti-neuropathic pain effects in the CCI neuropathic pain model. Abbreviations: AMG, aminoglutethimide, KM, koumine, MWT, mechanical withdrawal threshold. Data are presented as the mean ± SEM, ***p < 0.001 vs. the corresponding vehicle group. In the formalin test, ^##^ *p* < 0.01 versus the 10 mg/kg KM group, ^$$^ *p* < 0.01 versus the 0.5 mg/kg Ro5-4864 group, ^&&^ *p* < 0.01 versus the 0.5 mg/kg PK11195 group. In the CCI neuropathic pain model, ^###^ *p* < 0.001 versus the 7 mg/kg KM group, ^$$$^ *p* < 0.001 versus the 1.0 mg/kg Ro5-4864 group. The comparison between the two groups was analyzed by independent samples *t*-test, and multiple comparisons were compared by one-way ANOVA followed by LSD post hoc test. Each group consisted of 6–10 animals.

To test whether neurosteroids are involved in the analgesic effect of koumine, a neurosteroid synthesis inhibitor (AMG, 10 mg/kg) was used. In addition, the previous results of our research group showed that koumine could exert analgesic effect alone at high dose, showing allosteric agonist effects. Therefore, 10 mg/kg and 7 mg/kg koumine were selected in the formailin-induced inflammatory pain model and CCI model of neuropathic pain in this manuscript, respectively (Xiong et al., 2021). As shown in Figure 2, the one-way ANOVA revealed significant among-group differences in the biting time in phase II (F_7, 68_ = 8.170, *p* < 0.001, Figure 2A). Post hoc LSD multiple comparison tests showed that 0.5 mg/kg PK11195 and Ro5-4864 produced a significant antinociceptive effect in phase II, and these analgesic effects were reversed by AMG, which was consistent with previous findings (DalBo et al., 2004). The TSPO ago-PAM koumine 10 mg/kg alone also exerted a prominent analgesic effect; similarly, the analgesic effect of koumine was dependent on neurosteroids because AMG prevented the effect (Figure 2A). The analgesic mechanism of koumine associated with neurosteroids was also studied in the CCI-induced neuropathic pain model. Since PK11195 has no analgesic effect in the neuropathic pain model (Liu et al., 2016), only Ro5-4864 was selected as the orthosteric ligand in this model. The results also showed that AMG abolished the analgesic effects of Ro5-4864 and koumine (Figure 2B), indicating that the analgesic mechanism of koumine may be associated with neurosteroids.

## Discussion

In the present study, we report the *in vivo* evaluation of the allostery of koumine when orthosteric ligand PK11195 was used and preliminarily explore the possible analgesic mechanism of koumine. Koumine is an ago-PAM of PK11195-mediated analgesic effects at TSPO. Furthermore, the analgesic mechanism of the TSPO ago-PAM koumine may be associated with neurosteroids because AMG can abolish the analgesic effects of koumine in inflammatory and neuropathic pain models.

Allosteric modulator mainly include PAM, negative allosteric modulator and neutral allosteric ligand. Probe dependence is one of the main characteristics of allostery. For a given receptor, the allosteric interaction of the allosteric modulator depends on the probe used, and for different probes, the allosteric modulator may have a different allostery (Wootten et al., 2013). For example, TCN-201 is a negative allosteric modulator of glycine efficacy but a neutral allosteric ligand of glutamate activity at the N-methyl-D-aspartate receptor (Christopoulos et al., 2014). We recently studied the allostery of koumine with TSPO in a formalin-induced inflammatory pain model, collagen-induced arthritis model and CCI model in which Ro5-4864 was used (Xiong et al., 2021). The results showed that when koumine (an ineffective dose) was combined with Ro5-4864, it significantly enhanced the analgesic and anti-inflammatory effects of Ro5-4864. PK11195 also has analgesic effects in the formalin-induced inflammatory pain model, and the allostery of koumine when orthosteric ligand PK11195 is used remains to be clarified. In the current study, we found that koumine prominently augmented the PK11195-mediated analgesic effect in phase II but have no significant effect on phase I in a formalin-induced inflammatory pain model, manifesting that koumine is an ago-PAM of the PK11195-mediated analgesic effect at TSPO. In addition to PK11195 and Ro5-4864, TSPO also has other orthosteric ligands, such as cholesterol. The probe dependence of allostery between koumine and TSPO when other TSPO orthosteric ligands are used remains to be further studied.

Noteworthily, only 0.0008 and 0.5 mg/kg of PK11195 have analgesic effect in phase I, and the effect is not dose-dependent. Both our research group and other research groups have observed that the effects of TSPO orthosteric ligand (such as PK11195) in phase I are not dose-dependent (DalBo et al., 2004; Xiong et al., 2021). One possible explanation for this observation is that the pain in phase I and phase II may be different. Phase I may be due to immediate and direct effects on sensory receptors, in contrast, phase II may be due to an inflammatory response (Hunskaar et al., 1985; Rujjanawate et al., 2003). And this may also be one of the reasons why koumine shows different effects on phase I and phase II. Moreover, we also observed that koumine only enhanced the analgesic effect of 0.0008–0.02 mg/kg PK11195 but not 0.00016, 0.1 and 0.5 mg/kg PK11195 in the phase II of formalin-induced inflammatory pain model. This may be related to the limited enhancement effect of allosteric modulation, that is, when the concentration of orthosteric ligand is too low or too high, the function of orthosteric ligand in the absence or presence of ago-PAM has no significant difference. Similar phenomena have been observed by other research group (Ignatowska-Jankowska et al., 2015).

TSPO can impair the total steroidogenic output and plays a crucial role in neurosteroidogenesis. In addition, in the neuropathic pain model induced by spinal nerve ligation, Ro5-4864 can effectively inhibit mechanical allodynia and thermal hyperalgesia and the analgesic effect can be antagonized by AMG (Wei et al., 2013), suggesting that the TSPO ligand may exert analgesic effects through neurosteroids. Koumine is a specific ligand of TSPO that has a similar affinity to the typical TSPO ligand (Xiong et al., 2021). Preliminary studies by our research group have shown that koumine can increase the level of neurosteroids in the rat spinal cord of CCI neuropathy (Xu et al., 2012). Thus, we conjecture that the analgesic effect of koumine may also be related to neurosteroidogenesis. In this study, we found that the analgesic effects of Ro5-4864 or PK11195 in a formalin-induced inflammatory pain model and a CCI neuropathic pain model can be antagonized by AMG, which can also abolished the analgesic effect of koumine in these inflammatory and neuropathic pain models, indicating that the analgesic mechanism of koumine may be associated with neurosteroids.

Together, we investigated the allostery of koumine extracted from *G. elegans* Benth. *in vivo* when the orthosteric ligand PK11195 was used and found that the analgesic mechanism of the TSPO ago-PAM koumine may be associated with neurosteroids. At the same time, due to the limitations of pharmacological methods, our research cannot fully confirm the allosteric modulatory effect of koumine on TSPO, and structure determination as a definitive evidence of allosteric modulator has yet to be further carried out (Christopoulos et al., 2014; Xiong et al., 2021). Our data further prove the allostery in TSPO and provide a solid foundation for koumine to be used as a new clinical candidate drug to treat pain.

## Data Availability

The original contributions presented in the study are included in the article/Supplementary Material, further inquiries can be directed to the corresponding author.
